# Vitamin E biofortification: enhancement of seed tocopherol concentrations by altered chlorophyll metabolism

**DOI:** 10.3389/fpls.2024.1344095

**Published:** 2024-02-26

**Authors:** Ping Qin, Peng Chen, Yuanwei Zhou, Wei Zhang, Yunyun Zhang, Jingjing Xu, Lu Gan, Yingnan Liu, Jill Romer, Peter Dörmann, Edgar B. Cahoon, Chunyu Zhang

**Affiliations:** ^1^ National Key Laboratory of Crop Genetic Improvement and College of Plant Science and Technology, Huazhong Agricultural University, Wuhan, China; ^2^ Yichang Academy of Agricultural Science, Ministry of Agriculture and rural areas, Yichang, Hubei, China; ^3^ Industrial Crops Institute of Yunnan Academy of Agricultural Sciences, Ministry of Agriculture and rural areas, Kunming, China; ^4^ Department of Biochemistry and Center for Plant Science Innovation, University of Nebraska-Lincoln, Lincoln, NE, United States; ^5^ Lincang Agricultural Technology Extension Center, Lincang, Yunnan, China; ^6^ Institute of Molecular Physiology and Biotechnology of Plants (IMBIO), University of Bonn, Bonn, Germany

**Keywords:** chlorophyll synthase, homogentisate phytyltransferase, homogentisate, phytyl diphosphate, geranylgeranyl diphosphate, tocopherol

## Abstract

Homogentisate Phytyltransferase (*HPT*) catalyzes condensation of homogentisate (HGA) and phytyl diphosphate (PDP) to produce tocopherols, but can also synthesize tocotrienols using geranylgeranyl diphosphate (GGDP) in plants engineered for deregulated HGA synthesis. In contrast to prior tocotrienol biofortification efforts, engineering enhanced tocopherol concentrations in green oilseeds has proven more challenging due to the integral role of chlorophyll metabolism in supplying the PDP substrate. This study show that RNAi suppression of *CHLSYN* coupled with *HPT* overexpression increases tocopherol concentrations by >two-fold in Arabidopsis seeds. We obtained additional increases in seed tocopherol concentrations by engineering increased HGA production via overexpression of bacterial *TyrA* that encodes chorismate mutase/prephenate dehydrogenase activities. In overexpression lines, seed tocopherol concentrations increased nearly three-fold, and resulted in modest tocotrienol accumulation. We further increased total tocochromanol concentrations by enhancing production of HGA and GGDP by overexpression of the gene for hydroxyphenylpyruvate dioxygenase (HPPD). This shifted metabolism towards increased amounts of tocotrienols relative to tocopherols, which was reflected in corresponding increases in ratios of GGDP/PDP in these seeds. Overall, our results provide a theoretical basis for genetic improvement of total tocopherol concentrations in green oilseeds (e.g., rapeseed, soybean) through strategies that include seed-suppression of *CHLSYN* coupled with increased HGA production.

## 1 Introduction

Vitamin E tocochromanols are a class of fat-soluble antioxidants that contain a homogentisate (HGA)-derived aromatic head group linked to an isoprenoid-derived hydrocarbon tail. Vitamin E tocochromanols are comprised of tocopherols and tocotrienols that differ based on the saturation of the hydrocarbon tail: tocopherols are saturated and tocotrienols are tri-unsaturated (Hunter and Cahoon, 2007). Additionally, α, β, γ and δ forms of tocopherols and tocotrienols occur that have differing numbers or arrangements of methylation of the head group that affect their bioavailability (Netscher, 2007; Yang et al., 2020). Vitamin E biosynthesis occurs in photosynthetic organisms such as higher plants, algae, and cyanobacteria (Lichtenthaler, 1968; Sattler et al., 2004) and is present in a number of plant organs (Hunter and Cahoon, 2007).

Vitamin E is a required nutrient in human and animal diets and functions as an antioxidant that quenches free radicals derived from processes such as unsaturated fatty acid peroxidation (Warner et al., 2003). Vitamin E tocochromanols typically accumulate in seeds are components of seed oils that contribute to their oxidative stability by quenching free radicals arising from unsaturated fatty acid peroxidation (Grusak and DellaPenna, 1999; Kanwischer et al., 2005; Muñoz and Munné-Bosch, 2019). Vitamin E has also been widely incorporated in the food and cosmetic industries as a supplement for prolonging food stability and preventing UV and ozone skin damage (Thiele and Ekanayake-Mudiyanselage, 2007; Kmiecik et al., 2019).

**Figure 1:**
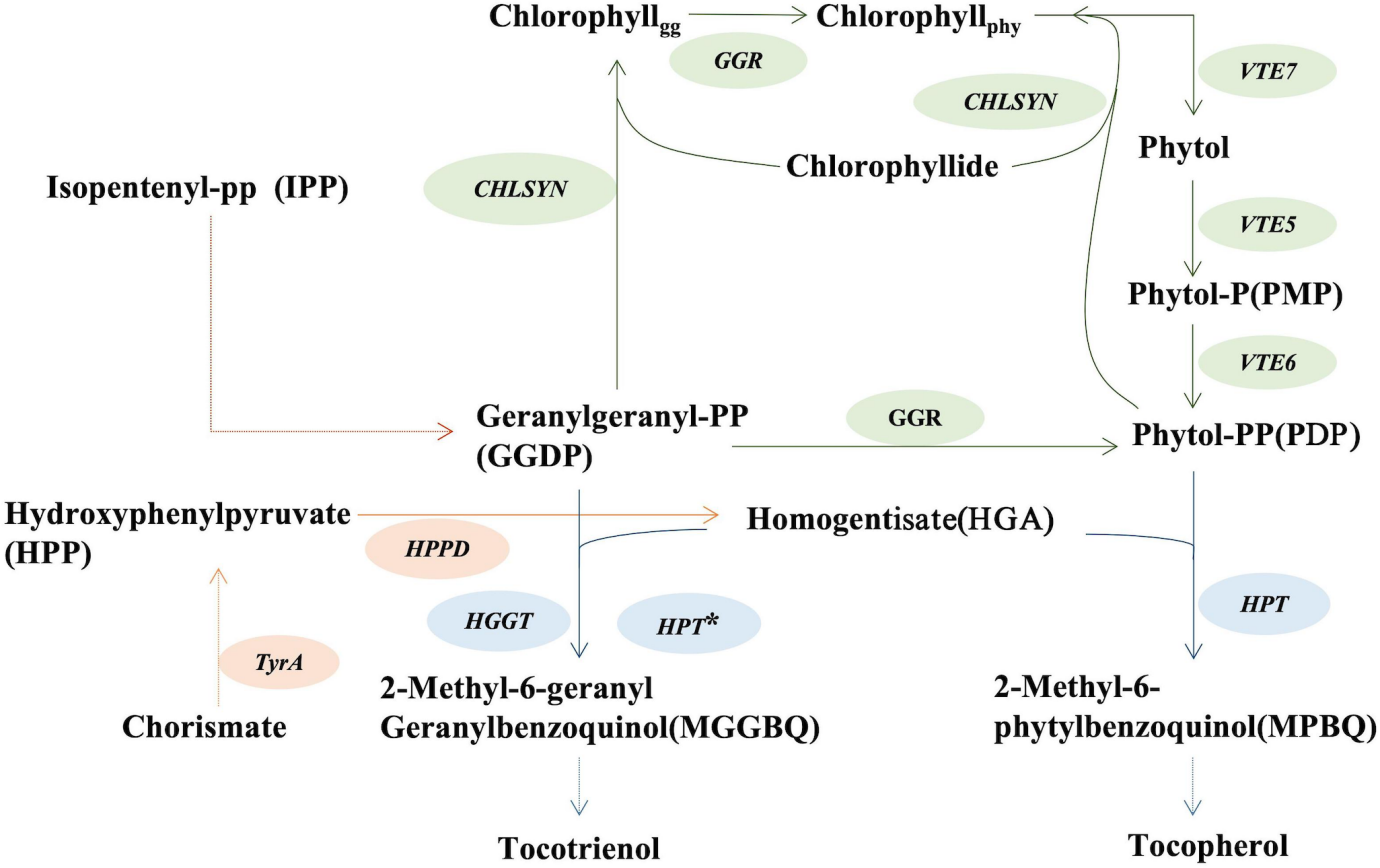
Schematic biosynthetic pathways of tocopherol and tocotrienol synthesis. *CHLSYN*, chlorophyll synthetase; *HPPD*, p-hydroxyphenylpyruvate dioxygenase; *HPT*, homogentisate phytyltransferase; *GGR*, geranylgeranyl reductase; *HGGT*, homogentisate geranylgeranyl transferase; *VTE5*, phytol kinase; *TyrA*, prephenate dehydrogenase. HPT*: When the endogenous level of homogentisate is high, *HPT* can also catalyze the step from GGDP to MGGBQ towards tocotrienol synthesis.

Given the nutritional and economic importance of vitamin E tocochromanols, considerable efforts have been directed toward their biofortification in oilseeds (Shintani and DellaPenna, 1998). Homogentisate geranylgeranyl transferase (HGGT) and homogentisate phytyltransferase (HPT or VTE2) are rate-limiting enzymes involved in the biosynthesis of tocotrienols and tocopherols, respectively, and have been the targets of research focused on tocochromanol production (Savidge et al., 2002; Cahoon et al., 2003; Rippert et al., 2004). *HGGT* was originally identified in seeds of monocots, including barley, wheat, and rice and is most active with geranylgeranyl diphosphate (GGDP) as its substrate (Cahoon et al., 2003; Yang et al., 2011; Zhang et al., 2013; Chen et al., 2017). Previous work has shown that heterologous expression of barley *HGGT* in soybean and corn leads to a six to tenfold increase in seed tocochromanols, principally in the form of tocotrienols (Cahoon et al., 2003; Konda et al., 2020). *HPT* primarily uses phytyl diphosphate (PDP) for tocopherol synthesis, but may also appropriate GGDP as a substrate for tocotrienol synthesis when the homogentisate level is high (**Figure 1**) (Cahoon et al., 2003; Collakova and DellaPenna, 2003; Yang et al., 2011; Zhang et al., 2013).

HGA, produced from the shikimic acid pathway, is substrate for both HGGT and HPT in the initial step of tocochromanol synthesis (Cahoon et al., 2003; Zhang et al., 2013) and is considered a limiting precursor for vitamin E production (Rippert et al., 2004; Karunanandaa et al., 2005; Yang et al., 2011; Stacey et al., 2016). Experiments aimed at generating large increases in HGA concentrations to enhance tocopherol production have resulted, instead, to the unexpected production of tocotrienols, with only small increases in tocopherol concentrations. This result is presumably due to limiting PDP pools for tocopherol biosynthesis that shifts the relative PDP : GGDP ratios to promote HPT-mediated tocotrienol biosynthesis. For example, the deregulated, enhanced HGA production by co-expression of transgenes for the yeast prephenate dehydrogenase and hydroxyphenylpyruvate dioxygenase (HPPD) in tobacco leaves yielded nearly no increase in tocopherol concentrations. This, instead, conferred production of tocotrienols and their accumulation to amounts ~ten-fold higher than tocopherols (Rippert et al., 2004). Similarly, overexpression of the *E. coli tyrA* gene and Arabidopsis *HPPD* gene generated large increases homogentisate concentrations in Arabidopsis leaves, which was accompanied by accumulation of tocotrienols, which are not normally present in Arabidopsis (**Figure 1**) (Zhang et al., 2013). Furthermore, co-overexpression of *HPPD*, *TyrA*, and *HPT* transgenes in transgenic canola and soybean seeds led to a two- to three-fold increase in total tocochromanol concentrations, primarily as tocotrienols rather than tocopherols (Karunanandaa et al., 2005).

The findings above highlight the feasibility of generating large increases in vitamin E tocochromanols, but these increases are largely the result of enhanced tocotrienol production. By contrast, biofortification of similar large increases in tocopherol content of plant organs has proven more elusive. It has succeeded in only more modest enhancement in tocopherol levels in plants. Findings from the Arabidopsis *vte5* mutant that is impaired in phytol kinase activity have indicated that PDP biosynthesis is a major limitation for tocopherol production in leaves and green seeds. These studies showed that nearly 80% of PDP required for tocopherol synthesis is derived from cycling and reduction of geranylgeraniol through chlorophyll (Valentin et al., 2005; Ischebeck et al., 2006; Zhang et al., 2015). Phytol formed from geranylgeranyl reductase activity using geranygeraniol on chlorophyll is released and converted to PDP by two sequential phosphorylation steps catalyzed by VTE5 and VTE6 kinases (Valentin et al., 2005; Gutbrod et al., 2019; Gutbrod et al., 2021). The resulting PDP is available for use in tocopherol biosynthesis. In Arabidopsis *vte5* mutants, seed tocopherol levels were reduced to 20% of those in wild type plants, suggesting that a large fraction of phytol generated through the chlorophyll turnover is used for PDP and subsequently tocopherol biosynthesis (Valentin et al., 2005; Ischebeck et al., 2006). The Arabidopsis *CHLSYN* knockout mutant had only 20-26% tocopherol in leaves with severe photosynthetic defects, suggesting a major portion of PDP for tocopherol synthesis comes from the chlorophyll salvage pathway (Vom Dorp et al., 2015; Zhang et al., 2015). Downregulation of *CHLSYN* by RNAi in Arabidopsis resulted in reduced chlorophyll content and higher levels of tocopherols in leaves, though tocochromanol levels in seeds were not determined (Zhang et al., 2015). This result was consistent with a negative correlation between *CHLSYN* expression levels and tocopherol content, in accordance with the competition for PDP between the chlorophyll and tocopherol biosynthetic pathways. downregulation of *CHLSYN* expression on tocopherol content in seeds may provide a route for enhancement of overall seed tocopherol concentrations.

In this study, we first found that the tocopherol content in Arabidopsis seeds is also negatively correlated with *CHLSYN* expression levels, as observed in *CHLSYN* overexpression and down-regulation lines. In a seed-specific *CHLSYN* RNAi background, we overexpressed *TyrA* and *HPT* with seed-specific promoters to stimulate tocopherol synthesis with high HGA input. We obtained Arabidopsis seeds displaying high vitamin E content primarily composed of tocopherols. We also found that elevated HGA production shifts the relative amount of PDP and GGDP and determines metabolic flow into tocopherol and tocotrienol biosynthesis. Overall, our findings provides a strategy for vitamin E biofortification of green oilseeds for enhanced tocopherol concentrations.

## 2 Materials and methods

### 2.1 Plant materials and growth conditions

Wild-type and transgenic *Arabidopsis thaliana* lines used for this study were of the Columbia-0 ecotype. Arabidopsis *vte5-2* seeds were previously described (Vom Dorp et al., 2015). Homozygous seeds carrying the 35S:*TyrA* and 35S:*AtHPPD*-35S:*TyrA* overexpression constructs were previously generated by our laboratory (Zhang et al., 2013). Plants were grown on plates with ½ MS agar supplemented with 2% (w/w) sucrose. Pot growth was performed in a growth chamber at 22°C under a 16 h day, with light intensity at 100 µmol m^-2^ sec^-1^.

### 2.2 Arabidopsis transformation

Arabidopsis plants were grown in a growth chamber in long day conditions. 4-5 weeks old healthy plants were chosen for Agrobacteria transformation using floral dip method (Clough and Bent, 1998; Zhang et al., 2015). The transformed plants were grown and seeds were harvested. Transgenic seeds were selected based on mCherry marker, followed by genotyping using gene-specific primers.

### 2.3 Vector construction and selection of transgenic plants

The vector pBinGlyRed3 containing a DsRed fluorescent protein marker under the control of the 35S promoter was used in this studies (Jach et al., 2001; Nguyen et al., 2013).

*CHLSYN* (AT3G51820) was amplified from Arabidopsis cDNA using primers as following: CHLSYN-F: 5’- GCTCTAGACCGTCGGTTCTATGACTTCGAT-3’ and CHLSYN-R: 5’- CCCCTCGAGTCAAAATACGCCTTTTTCAGT-3’ (restriction sites underlined). All PCR reactions were performed using Phusion polymerase (Vazyme, Wuhan, China). The PCR product was cloned into pBinGlyRed3 using *Xba*I/*Xho*I sites. The resulting plasmid with *AtCHLSYN* flanked by glycinin promoter and glycinin terminator was designated as SYN-OE.

A specific *AtCHLSYN* 430bp fragment was chosen for constructing the *CHLSYN* RNAi (RNA interference) vector. The forward segment Si01 was amplified using the following primers: Si01-F, 5’-CCGCTCGAGGACGCAATTAATGAGCCATATCG-3’ and Si01-R, 5’-GGACTAGTTGCCAAAAGCTACTGGGAGAGAC-3’. A second fragment Si02 was amplified using Si02-F: 5’-GCTCTAGAGACGCAATTAATGAGCCATATCG-3’ and Si02-R: 5’-CCCAAGCTTTGCCAAAAGCTACTGGGAGAGA-3’. The Si01 segment was cloned into the pINTRON vector using *Xho*I/*Bcu*I sites. Into the resulting vector pIN-Si01 the second Si02 segment was ligated using *Hind*III/*Xba*I sites (Nguyen et al., 2013). The resulting vector pIN-Si containing a *Not*I fragment with Si01-intron-Si02 was subcloned into vector pBetaCon with glycinin promoter (seed-specific promoter) and phaseolin terminator. Finally, the complete expression cassette (glycinin promoter:Si01-intron-Si02::phaseolin terminator) was sub-cloned into pBinGlyRed3 using the *Sgs*I site. The final interference vector pBinGlyRed3-*CHLSYN*: RNAi was abbreviated as SYN-RNAi.

For *HPT* overexpression, *HPT* (AT2G18950) cDNA was amplified from Arabidopsis cDNA using the following primers: At*HPT*-*EcoR*I-LP, 5’-CCGAATTCTCACTTCAAAAAAGGTAACAG-3’; At*HPT*-*Sma*I-RP, 5’-TTCCCGGGATGGAGTCTCTGCTCTCTAGT-3’ (restriction sites underlined). The PCR product was cloned into pBinGlyRed3 with *EcoR*I/*Sma*I sites, the resulting vector pBinGlyRed3-35S:At*HPT* was designated 35S:At*HPT*.

The SgsI fragment from 35S:At*HPT* containing a complete cassette was inserted into the *Sgs*I site of pBinGlyRed3 and the *Mlu*I site of Red3-Si to construct pBinGlyRed3 –Ole : At*HPT* (OleAtHPT) and pBinGlyRed3-*CHLSYN*: RNAi + Ole-HPT. Primer Red3-BamHI-F GCGTATGGATTATGGAACTATCA and AtHPT-YZ-R AAAGGAGATATATCAGAAACCTTCTC were used to confirm the transcription orientation of the two cassettes.

### 2.4 HPLC analysis of tocochromanol content and composition

An HPLC with fluorescence detector was used for quantification of seed tocochromanol contents, using 5,7-dimethyltocol as standard (Yang et al., 2011). ~5 mg of dried seeds, 1 ml methanol/dichloromethane (9:1 v/v) and 5 μl internal standard 5,7-dimethyltocol (Matreya, www.matreya.com) were added to a 2 ml centrifuge tube, the sample was ground with steel balls, then incubate at room temperature for 3 h and centrifuged. Tocochromanols were separated on an Agilent Eclipse XDB-C18 reversed-phase column (4.6 × 150 mm, 5 μm particle size, www.agilent.com), with isocratic conditions of methanol/water (95:5 v/v) at flow rate of 1.5 ml/min. The abundance of each compound was monitored by excitation at 292 nm and emission at 330 nm.

### 2.5 Semi-quantitative PCR analysis

Total RNA was extracted from seeds harvested 10 days after flowering, with the TRIzol Reagent Kit (Ambion) according to the manufacture’s protocol. RNA was reverse transcribed to cDNA using the HiScript II 1st Strand cDNA Synthesis Kit (Vazyme). qRT-PCRs was performed with the SYBRgreen qPCR Master Mix (Vazyme) using the CFX Connect™ real-time PCR detection system (BIO-RAD, Hercules, CA, USA). The following qRT primers were used: qAtHPT-F1-1, 5’-TCGCAAAACCGAAGTTTAGGAAC-3’; qAtHPT-R1-1, 5’-TGTTTGCTATTCGAGTCGAAAGC-3’ for AtCHLSYN. Actin7 (AT5G09810) was chosen as reference gene, qRT primers: βActin7-F, 5’-GATATTCAGCCACTTGTCTGTGAC-3’; and βActin7-R: 5’-CATGTTCGATTGGATACTTCAGAG-3’.

### 2.6 Quantification of GGDP, PDP and HGA contents in seeds

Mature Arabidopsis seeds were used for GGDP and PDP determination (Valentin et al., 2005). Prenyl-transferase assays were based on a previously described method (Collakova and DellaPenna, 2003; Yang et al., 2011). UPLC LC-MS system linked to a QTRAP4500 mass spectrometer was used for the determination of HGA, the method is described by Karunanandaa et al. (2005).

### 2.7 Statistical analysis

We have quantified the seed VitE contents in transgenic materials with single-copy T-DNA insertion based 3:1 segregation ratio of the mCherry marker in T2 generation to avoid gene dosage effect. All transgenic lines have their corresponding non-transgenic control. Due to the fluctuation of seed VitE contents by environmental factors, the tocopherol contents were normalized to 540 µg/g as “wild type” value based on literature (Karunanandaa et al., 2005) and our empirical Col.0 seed records under conditions used in this study. Positive transgenic seeds (Red) and negative segregant seeds of Ole : HPT/Col-0, (SYN-RNAi+Ole : HPT)/Col-0 and (SYN-RNAi+Ole : HPT)/35sTyrA transgenic lines were separated according to their segregation ratio of 3:1 in the T_2_ generation. According to this, we calculated the seed tocochromanol contents in T2 generation based on seeds with red fluorescence accounting for 75% of the total T2 seeds and non-transgenic seeds accouting for 25% from a heterozygous T1 plant.


The tocochromanols of T2 seeds=levels from red (transgenic) seeds × 75%+ levels from non-transgenic seeds × 25%

## 3 Results

### 3.1 Chlorophyll synthase expression levels and seed tocopherol content are negatively correlated

**Figure 2:**
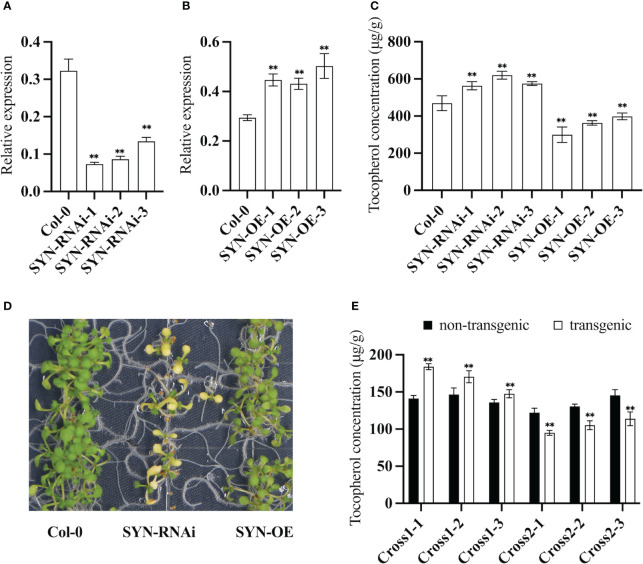
Expression pattern of *AtCHLSYN* and quantification of seed tocochromanol contents in Arabidopsis transgenic lines. **(A, B)**, qRT-PCR of *AtCHLSYN* expression in SYN–RNAi (RNAi line of *AtCHLSYN*) or SYN (overexpression line of *AtCHLSYN*) transgenic seeds (10 days after flowering). **(C)** Tocopherol contents in T2 mature seeds of SYN-RNAi and SYN transgenic plants. **(D)** Growth phenotype of young seedlings of SYN-RNAi transgenic plants, SYN and Col-0. **(E)** *vte5-2* homozygous mutants crossed with SYN-OE and SYN-RNAi transgenic lines, cross1 and cross2 represent F3 plants of *vte5* carrying SYN-OE and SYN-RNAi constructs respectively. Tocopherol contents of seeds with DsRed fluorescence (transgenic) and seeds lacking DsRed fluorescence (non-transgenic) from F3 progeny. Seed tocopherol contents were calculated from three biological replicates. ** represents significance level of p < 0.01 by student t-test.

RNAi lines of *CHLSYN* were generated and annotated as “SYN-RNAi” (**Figures 2A, C**). The T-DNA in the expression vector for these lines contained a DsRed selection marker to facilitate the screening procedure for transgenic events. From 68 T_1_ positive lines, we isolated 3 heterozygous lines from the T_2_ generation with a single copy of the SYN-RNAi insertion. qRT-PCR results indicated that RNAi lines had reduced transcript levels of the *CHLSYN* gene (**Figure 2A**). The SYN-RNAi seeds upon maturation had higher tocochromanol contents, with up to 34% increases of tocopherol concentrations compared to amounts in non-transgenic segregant seeds (**Figure 2C**). *AtCHLSYN* was also overexpressed under the control of the strong seed-specific glycinin promoter. Positive transgenic lines were selected and annotated as “SYN-OE” (Zhang et al., 2015). We obtained a total of 54 SYN positive lines in the T_1_ generation, and picked 3 single-copy insertion T_2_ plants in the heterozygous state based on their 3:1 segregation ratio through DsRed marker selection. qRT-PCR results indicated that SYN-OE lines had higher transcript levels of the *CHLSYN* gene than control (**Figure 2B**). In the T_2_ generation, lines with seed-specific over-expression of *CHLSYN* showed large reduction in tocopherol content, with reductions of ≤42% compared to levels in isogenic wild type segregant seeds (**Figure 2C**). Young seedlings of SYN-RNAi were yellow but gradually turned green during later development, whereas the SYN-OE seedlings were morphologically similar to wild type plants (**Figure 2D**). Collectively, results from the SYN-OE and SYN-RNAi transgenic lines suggest that transcript levels of *CHLSYN* are negatively correlated with seed tocopherol contents in Arabidopsis transgenic plants.

*VTE5* catalyzes the conversion of phytol to PMP during chlorophyll degradation (**Figure 1**). We investigated whether a negative correlation also exists when the level of PDP is low. For these studies, the *vte5* mutant, which has altered PDP accumulation was used. We crossed the *vte5* mutant with the SYN-RNAi or SYN-OE transgenic plants and measured the seed tocopherol concentrations of the progeny. Measurements were conducted with the homozygous state for *vte5* (knock-out background) and a heterozygous state for SYN-OE or SYN-RNAi to investigate the effect of up- or down- regulation of *CHLSYN* (**Figure 2E**). As shown in **Figure 2**, average tocopherol concentrations in non-transgenic *vte5* seeds was 140 μg/g, a reduction in tocopherol concentrations compared to wild type seeds (400~500 μg/g, **Figure 2C**). Knocking out *VTE5* dramatically decreased the tocopherol content to ~20% of that in wild type. Despite this, a negative correlation could still be observed between *CHLSYN* transcript level and tocopherol content in engineered *vte5* seeds (**Figure 2E**).

### 3.2 Modest increase in tocopherol content in seeds of transgenic Ole : AtHPT and AtCHLSYN RNAi plants

**Figure 3:**
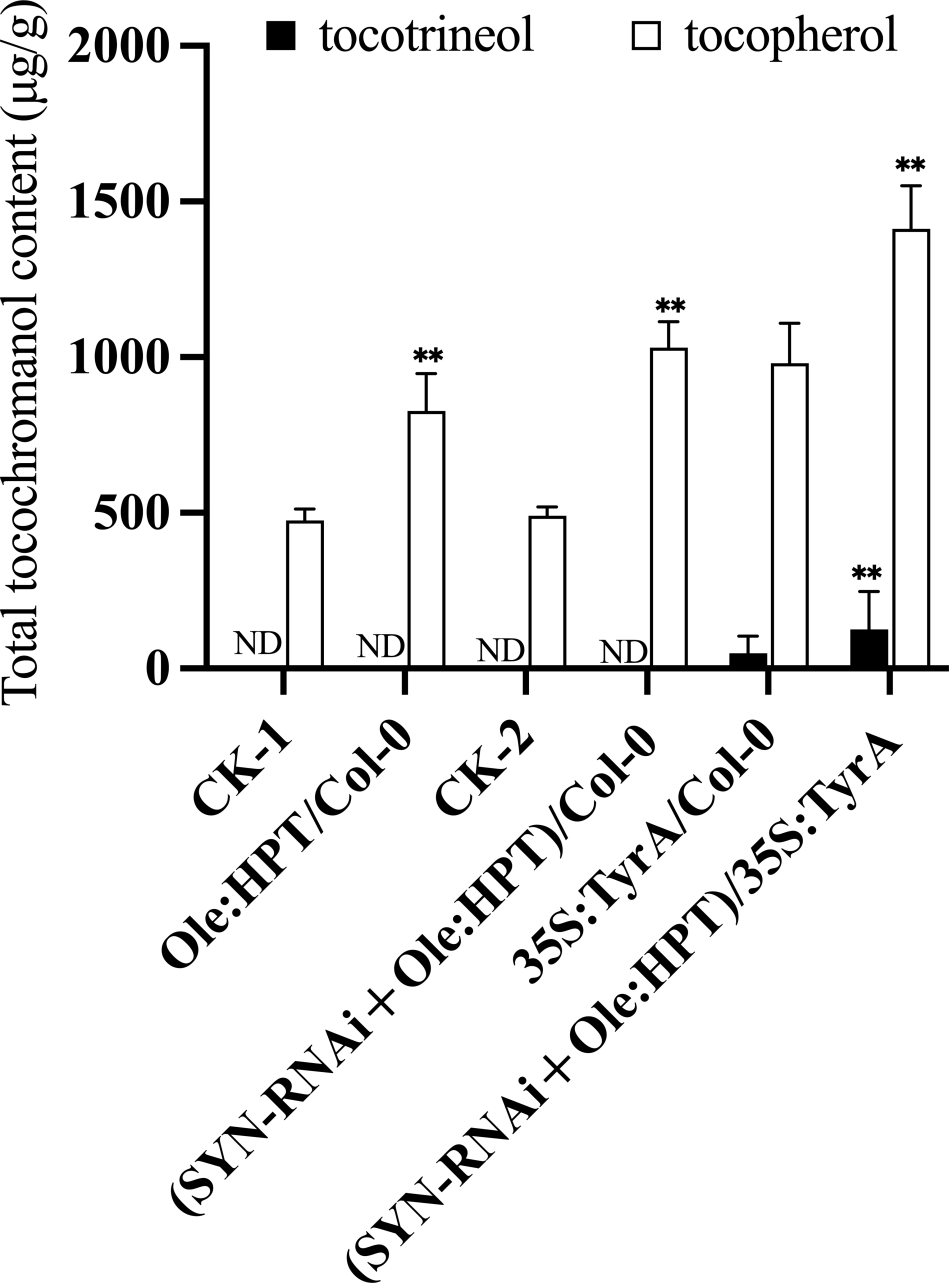
Synergistic role of HPT and TyrA overexpression in combination with SYN-RNAi on seed tocopherol or tocotrienol accumulation. Tocochromanol contents in mature T2 seeds from Col-0 transformed with Ole : HPT construct (Ole : HPT/Col-0) and SYN-RNAi+Ole : HPT construct ((SYN-RNAi+Ole : HPT)/Col-0) respectively. CK-1 and CK-2 represent the separated seeds lacking DsRed fluorescence from Ole : HPT/Col-0 and (SYN-RNAi+Ole : HPT)/Col-0 transgenic plants. Tocochromanol contents of seeds from Col-0 transformed with 35S:TyrA construct (35S:TyrA/Col-0) and 35S:TyrA/Col-0 transformed with SYN-RNAi+Ole : HPT construct. Seed tocochromanol contents were calculated from three biological replicates. ND, below the limit of detection. ** represents significance level of p < 0.01 by student t-test.

HPT is a rate-limiting enzyme for tocopherol biosynthesis (**Figure 1**). Under most conditions, this enzyme only catalyzes the condensation of PDP with HGA during tocopherol synthesis, but in the presence of high levels of homogentisate, the HPT enzyme may also resort to using GGDP for condensation with HGA during tocotrienol synthesis (Yang et al., 2011). Utilizing the seed-specific oleosin promoter to drive *AtHPT* expression, seed tocochromanol contents in transgenic plants were measured (**Figure 3**). After two generations of selfing from the T_1_ positive transgenic lines, 13 T_2_ lines with a single copy insertion of Ole : *AtHPT* (Ole : HPT/Col-0) were obtained (**Figure 3**). These seeds had ~1.8 times the seed tocopherol concentration relative to wild type segregants (**Figure 3**, **Supplementary Table 1**). On the basis of this result, we further combined SYN-RNAi with Ole : HPT/Col-0 in a single construct that was transferred into transgenic plants (**Figure 3**). As shown in **Figure 3**, the seed tocopherol concentration in (SYN-RNAi+Ole : HPT)/Col-0 plants was ~2.1 times higher than that in non-transgenic segregant plants (**Figure 3**, **Supplementary Table 2**). The seeds of (SYN-RNAi+Ole : HPT)/Col-0 plants revealed a higher seed tocopherol content than seeds overexpressing the *HPT* gene alone. These data suggest that the SYN-RNAi transgene may dramatically reduce the *CHLSYN* activity for PDP recycling, and allow more PDP to be available for tocopherol biosynthesis.

### 3.3 Seed tocopherol content was increased in seeds with CHLSYN RNAi background carrying Ole : AtHPT and 35S:TyrA constructs

HGA along with PDP is substrate for tocopherol synthesis and is formed from the shikimic acid pathway (Raclaru et al., 2006). Overexpression of *TyrA* has been shown to effectively increase HGA content in Arabidopsis seeds (Karunanandaa et al., 2005). We introduced the (SYN-RNAi+Ole : HPT)/Col-0 construct into a plant homozygous for 35S:TyrA/Col-0. We annotated these lines as “(SYN-RNAi+Ole : HPT)/35S:TyrA”. Transgenic T_2_ plants with single copy insertions were used for seed tocochromanol content determination (**Figure 3**). We found that the tocopherol content of non-red seeds (carrying 35S:TyrA only) were 1.7 times of wild type seeds on average (**Figure 3**, **Supplementary Table 3**), and tocopherol content of red seeds (carrying (SYN-RNAi+Ole : HPT)/35S:TyrA) were ≤2.7 times those of wild type seeds (**Figure 3**, **Supplementary Table 3**). The maximum tocopherol content of (SYN-RNAi+Ole : HPT)/35S:TyrA-9 seeds was or ~1,600 µg/g seed wt or 2.7 times those of the wild type seed concentrations. In our knowledge, this is the highest reported accumulation of tocopherols in Arabidopsis seeds. In contrast, low amounts of tocotrienols were detected in red seeds (carrying (SYN-RNAi+Ole : HPT)/35S:TyrA) and non-red seeds (carrying 35S:TyrA only), with the average tocotrienol content being 8% and 5% of total tocochromanol, respectively (**Figure 3**, **Supplementary Table 3**). The RNA interference of the *CHLSYN* gene did not alter the fatty acid content and composition of Arabidopsis seeds in the wild type, 35S:TyrA/Col-0 and (SYN-RNAi+Ole : HPT)/Col-0 backgrounds (**Supplementary Table 4**).

**Figure 4:**
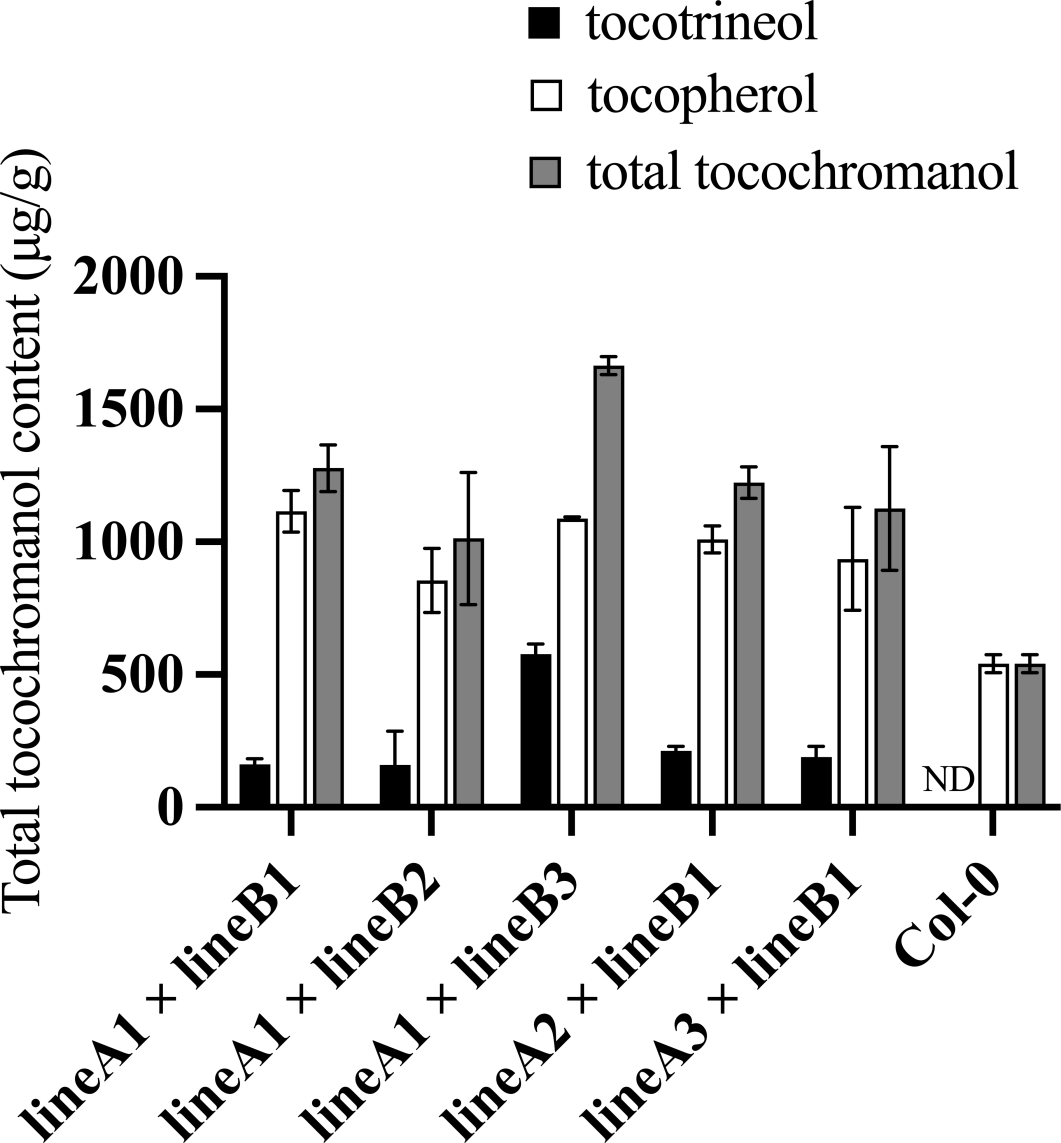
Measurements of total tocochromanol contents in seeds harboring the combination of (35S:TyrA+35S:HPPD)/Col-0 and (SYN-RNAi+Ole : HPT)/Col-0 constructs. Total tocochromanol contents from F2 seeds from crosses between lineA: (35S:TyrA+35S:HPPD)/Col-0 and lineB: (SYN-RNAi+Ole : HPT)/Col-0. ND, below the limit of detection.

*TyrA-*encoded bifunctional chorismate mutase/prephenate dehydrogenase catalyzes the conversion of chorismate to HPP. HPPD catalyzes the conversion of HPP to HGA (**Figure 1**). A transgenic Arabidopsis plant with *HPPD* and *TyrA* over-expression constructs contains more available HGA than lines with 35S:TyrA/Col-0 only (Zhang et al., 2013). Reciprocal crossing was performed using (35S:TyrA+35S:HPPD)/Col-0 plants with the above (SYN-RNAi+Ole : HPT)/Col-0 plants (**Figure 4**). In crosses with (35S:TyrA+35S:HPPD)/Col-0-2-4 as maternal parent but different paternal (SYN-RNAi+Ole : HPT)/Col-0 lines, we observed altered tocopherol and tocotrienol concentrations (**Figure 4**), suggesting that the expression levels of *CHLSYN* and *HPT* influence seed vitamin E synthesis. On the other hand, when (SYN-RNAi+Ole : HPT)/Col-0 was fixed but different (35S:TyrA+35S:HPPD)/Col-0 lines used as paternal parent, no major difference was observed (**Figure 4**), suggesting that *HPT* and *CHLSYN* are dominating factors over *HPPD* and *TyrA* for seed tocopherol and tocotrienol synthesis.

We further measured tocochromanol contents in the seeds of the F_1_ progeny. In the crosses, using (35S:TyrA+35S:HPPD)/Col-0 as paternal parent and (SYN-RNAi+Ole : HPT)/Col-0 as maternal recipient, a maximum of 1664 μg/g total tocochromanol was observed in F_1_ seeds of (35S:TyrA+35S:HPPD)/Col-0-2-4 x (SYN-RNAi+Ole : HPT)/Col-0-3. The total tocochromanol, which includes considerable amount of tocotrienols (35%) is equivalent to 3.1 times that of the wild type control (**Figure 4**, **Supplementary Table 5**). Another reciprocal combination (SYN-RNAi+Ole : HPT)/Col-0-1 ×(35S:TyrA+35S:HPPD)/Col-0-2-4, resulted in slightly lower total tocochromanol but considerably less tocotrienol content (11%, **Supplementary Table 5**). In the F_2_ population of (TyrA+HPPD)-OE-2-4 x (SYN-RNAi+Ole : HPT)/Col-0-3 and (SYN-RNAi+Ole : HPT)/Col-0-1 ×(TyrA+HPPD)-OE-2-4, the maximum seed tocochromanol concentration was 1953 µg/g, which is 3.4 times that of wild type control (**Supplementary Table 6**). Seed tocopherol concentrations ranging from 1.9 to 2.4 those that of wild type seeds Tocotrienols accounted for 10% to 30% of the total seed tocochromanol (**Supplementary Table 6**). Overexpression of the *HPPD* gene in the (SYN-RNAi+Ole : HPT)/Col-0-1×35S:TyrA background further increased the total content of tocochromanols, but the content of tocopherol was slightly reduced.

The tocochromanol content of red fluorescent seeds and non-red seeds were separately measured in the T_2_ population. In order to compare our results to those of the Karunanandaa et al., 2005 studies, the values in this study were adjusted to the same level (**Table 1**). The converted results show that the average tocopherol content of (SYN-RNAi+Ole : HPT)/35S:TyrA line was 2.2 times as high as those of the wild type, providing a significant improvement compared to the Napin : HPT+Napin : TyrA line and the Napin : HPPD+Napin: TyrA+Napin : HPT line (Karunanandaa et al., 2005). The average seed tocotrienol of these prior studies was further increased to 54% of the total tocochromanols upon introducing Napin : HPPD due to a higher level of HGA (Savidge et al., 2002; Karunanandaa et al., 2005). In our study, the proportion of tocotrienols was significantly lower than results from Karunanandaa et al., 2005. By inhibiting the expression of the *CHLSYN* gene, the average seeds tocotrienol contents vary from 8% to 19% in (SYN-RNAi+Ole : HPT)/35S:TyrA line and (SYN-RNAi+Ole : HPT)/Col-0 x (35S:TyrA+35S:HPPD)/Col-0 line (**Table 1**).

**Table 1:** Comparison of seed tocopherol content between this study and prior results.

Transgenic lines	Tocopherols				Tocotrienols			
	Maximum (µg/g)	Fold	Average (µg/g)	Fold	Maximum (µg/g)	Maximum percentage	Average (µg/g)	Average percentage
Current study								
Ole : HPT/Col-0 ^a^	929	1.7x	848	1.6x	0	0%	0	0%
(SYN-RNAi+Ole : HPT)/Col-0 ^b^	1195	2.2x	989	1.8x	0	0%	0	0%
(Ole : HPT+SYN-RNAi)/35S:TyrA ^c^	1344	2.5x	1186	2.2x	244	16%	97	8%
(SYN-RNAi+Ole : HPT)/Col-0 x (35S:TyrA+35S:HPPD)/Col-0 ^d^	1319	2.4x	1158	2.1x	498	27%	271	19%
Karunanandaa et al. (2005)								
Napin : HPT+Napin : TyrA ^e^	1179	2.2x	860	1.6x	279	23%	120	12%
Napin : HPPD+Napin : TyrA +Napin : HPT ^f^	1022	1.9x	702	1.3x	1688	62%	834	54%

a, b, c: Data from T_2_ population.

d: Data from F_2_ population.

e,f: Data from Karunanandaa et al. (2005).

### 3.4 High HGA and GGDP led to the tocotrienol biosynthesis in Arabidopsis seeds

**Figure 5:**
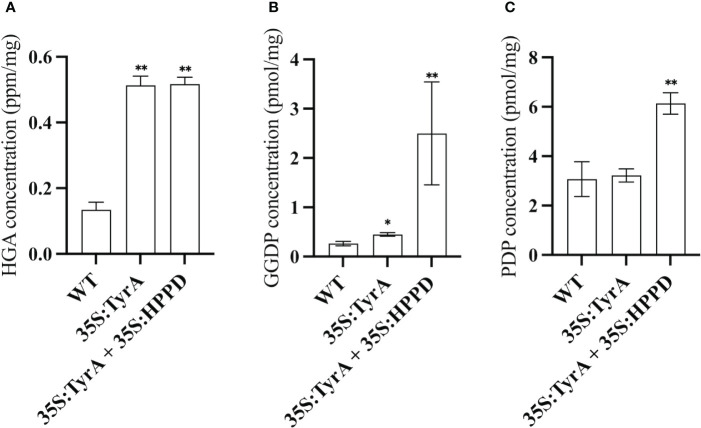
Measurement of HGA **(A)**, GGDP **(B)**, PDP **(C)** contents in seeds of 35S:TyrA/Col-0, 35S:TyrA+35S:HPPD plants. Data calculated from three biological replicates, * and ** indicate significant differences at p < 0.05 and p < 0.01 respectively, by Student’s t-test.

Based on the results above, it is observed that genes from different metabolic pathways can be used together to boost seed vitamin E content. When comparing the vitamin E contents and compositions between (SYN-RNAi+Ole : HPT)/35S:TyrA and (SYN-RNAi+Ole : HPT)/Col-0 x (35S:TyrA+35S:HPPD)/Col-0, a substantial amount of tocotrienol was observed in the latter case. In order to verify the synthesis of tocotrienol, we quantified PDP, GGDP and HGA contents in mature seeds of 35S:TyrA/Col-0 and (35S:TyrA+35S:HPPD)/Col-0 (**Figure 5**). High HGA contents were detected in two transgenic seeds with similar level (**Figure 5A**). We also found that GGDP levels in transgenic seeds were increased in various degrees, the improvement of GGDP contents in (35S:TyrA+35S:HPPD)/Col-0 seeds were ten-fold than those in wild type seeds, it is far greater than the 35S:TyrA/Col-0 seeds (**Figure 5B**). Our results from seeds confirm the conclusion that a high level of HGA and GGDP concentration lead to the tocotrienol synthesis in seeds. PDP levels of (35S:TyrA+35S:HPPD)/Col-0 seeds were doubled increased than wild type, rather than 35S:TyrA/Col-0 seeds (**Figure 5C**). We hypothesize that high GGDP contents might be the reason for the improvement of PDP, but high GGDP/PDP ratios is unfavorable to the synthesis of tocopherols.

## 4 Discussion

**Figure 6:**
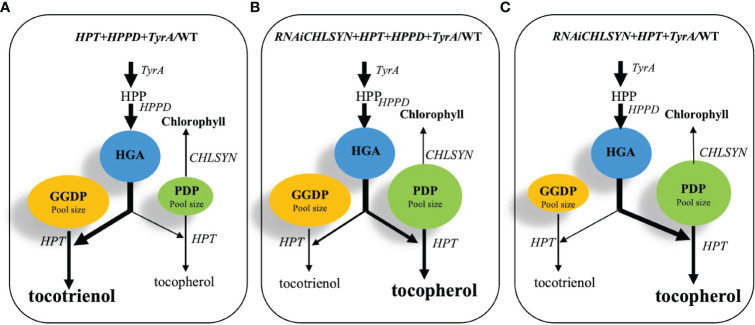
Schematic model for the metabolic engineering of *CHLSYN*, *TyrA*, *HPPD* and *HPT* genes to achieve high seed tocochromanol contents in Arabidopsis. **(A–C)**, three scenarios of combinations of different genetic backgrounds for the chlorophyll salvage pathway, the *TyrA* and *HPPD* pathway regulating HGA content, and the *HPT* expression level alter the balance between GGDP and PDP, resulting in tocotrienol or tocopherol synthesis, respectively. The thickness of the arrows represents flux through the metabolic pathway; the sizes of the blue, green and yellow circles showing HGA, PDP and GGDP contents were adjusted according to the metabolite pool size under each scenario. The dominating product of the pathway, i.e. tocopherol or tocotrienol, were shown in bold letters. According to the results, **(B)** scenario would be the best option, resulting in elevated PDP and HGA levels, as well as a favored condition for the tocopherol biosynthesis.

In this work, we used biotechnological approaches to increase total Arabidopsis seed tocochromanol concentrations, and in particular, tocopherol concentrations. First, our results confirmed that the negative correlation between *CHLSYN* expression and tocochromanol synthesis, previously observed in Arabidopsis leaves, also occurs in seeds. Second, we achieved the highest report tocopherol concentrations in mature Arabidopsis seeds via genetic combination of Ole : HPT, 35S:TyrA/Col-0 and SYN-RNAi (**Table 1**). In the transgenic lines with *TyrA* and *HPPD* overexpression, the resulting high HGA and GGDP input triggered HPT activity to use GGDP as a substrate for tocotrienol synthesis (**Figure 6**). In combination with SYN-RNAi, which increases the pool size of PDP available for tocopherol synthesis, HPT-OE and 35S:TyrA/Col-0 create a genetic background favorable for high accumulation of tocopherol, resulting in a maximum of 2.5 times the seed tocopherol elevation compared to WT (**Table 1**).

A key conclusion from this study is the negative correlation between chlorophyll synthase (*CHLSYN*) expression levels and tocopherol concentrations in Arabidopsis seeds. Indeed, we propose that the metabolic flow from either GGDP or PDP to tocotrienol or tocopherol synthesis, respectively, is critical for the final proportion of tocochromanols in mature seeds (**Figure 6**). Our data suggests that starting from the background of *CHLSYN* downregulation and *HPT* overexpression, simultaneous overexpression of *HPPD* and *TyrA* resulted in an optimal accumulation of total tocochromanol and tocopherols (**Figure 6B**). The overexpression of *HPPD* and *TyrA* boosts HGA supply and therefore increases the pool size of GGDP and PDP (**Figure 5**, **Figures 6A, B**). Furthermore, at high levels of HGA, *HPT* reveals a tendency toward using both GGDP and PDP for tocotrienol and tocopherol synthesis, respectively. Introduction of the *CHLSYN* RNAi construct results in the increased availability of PDP for tocopherol synthesis. Together with the elevated pool sizes of both GGDP and PDP, the balance shifts towards tocopherol production resulting in high seed tocopherol accumulation (**Figure 6B**). As seed tocopherol accumulation is a highly dynamic process, it would be useful to quantify the level of PDP and GGDP at different stages of seed development. However, the technique of PDP and GGDP measurement in minute amounts of seed material is not currently available. Although we could not observe a significant change of the PDP and GGDP pools in CHLSYN-RNAi or CHLSYN-OE seeds, the effect from (35S:TyrA+35S:HPPD)/Col-0 is apparent (**Figure 5**). Pool sizes at seed maturation reflect biosynthetic ability during seed development. Accordingly, we propose that the content and ratio of GGDP and PDP are critical for metabolic flow toward the synthesis of tocotrienols or tocopherols, as previously suggested (Yang et al., 2011; Zhang et al., 2013).

The genetic manipulations reported in this study can be theoretically used for the improvement of tocopherol concentrations in any photosynthetic “green” oilseed crop, including canola and soybean. While most studies on oilseeds have achieved significant increases in tocotrienol concentrations, enhancing tocopherol accumulation has been more elusive. Our current work demonstrates that RNAi of *CHLSYN* in the chlorophyll salvage pathway can reduce the final proportion of tocotrienols in total seed tocochromanols. We proposed a scenario in which overexpression of *TyrA* and *HPT* combined with *CHLSYN* suppression created a condition that favors tocopherol biosynthesis (**Figure 6B**). These data will provide important information to guide future genetic engineering for increasing seed tocopherol contents in oil seed crops. Since many crop species, e.g. *Brassica napus*, are polyploid, in contrast to *Arabidopsis*, the numbers of *CHLSYN* homologs are higher. Therefore, the use of CRISPR technology would be instrumental in generating mutants for disrupting some of the multiple *CHLSYN* loci for increasing PDP input. Based on these mutant backgrounds, suitable genetic material accumulating elevated amounts of tocopherols in mature seeds could be obtained by overexpressing *TyrA* and *HPT*.

## Data availability statement

The original contributions presented in the study are included in the article/**Supplementary Material**. Further inquiries can be directed to the corresponding authors.

## Author contributions

CZ: Conceptualization, Funding acquisition, Resources, Writing – review & editing. PQ: Conceptualization, Data curation, Formal Analysis, Investigation, Methodology, Project administration, Software, Supervision, Writing – original draft, Writing – review & editing. PC: Conceptualization, Formal Analysis, Investigation, Project administration, Resources, Software, Validation, Visualization, Writing – original draft, Writing – review & editing. YWZ: Writing – review & editing. WZ: Conceptualization, Data curation, Formal Analysis, Writing – review & editing. YYZ: Formal Analysis, Investigation, Methodology, Writing – review & editing. JX: Writing – review & editing. LG: Funding acquisition, Resources, Visualization, Writing – review & editing. YL: Investigation, Validation, Visualization, Resources. JR: Investigation, Writing – review & editing, Data curation, Formal Analysis, Methodology. PD: Data curation, Formal Analysis, Investigation, Methodology, Writing – review & editing. EC: Writing – review & editing.
